# Selection of reliable reference genes for gene expression studies in peach using real-time PCR

**DOI:** 10.1186/1471-2199-10-71

**Published:** 2009-07-20

**Authors:** Zhaoguo Tong, Zhihong Gao, Fei Wang, Jun Zhou, Zhen Zhang

**Affiliations:** 1 College of Horticulture, Nanjing Agricultural University, 1 Tongwei Road, Weigang, Nanjing 210095, PR China

## Abstract

**Background:**

RT-qPCR is a preferred method for rapid and reliable quantification of gene expression studies. Appropriate application of RT-qPCR in such studies requires the use of reference gene(s) as an internal control to normalize mRNA levels between different samples for an exact comparison of gene expression level. However, recent studies have shown that no single reference gene is universal for all experiments. Thus, the identification of high quality reference gene(s) is of paramount importance for the interpretation of data generated by RT-qPCR. Only a few studies on reference genes have been done in plants and none in peach *(Prunus persica *L. Batsch). Therefore, the present study was conducted to identify suitable reference gene(s) for normalization of gene expression in peach.

**Results:**

In this work, eleven reference genes were investigated in different peach samples using RT-qPCR with SYBR green. These genes are: actin 2/7 (*ACT*), cyclophilin (*CYP2*), RNA polymerase II (*RP II*), phospholipase A2 (*PLA2*), ribosomal protein L13 (*RPL13*), glyceraldehyde-3-phosphate dehydrogenase (*GAPDH*), 18S ribosomal RNA (*18S rRNA*), tubblin beta (*TUB*), tubblin alpha (*TUA*), translation elongation factor 2 (*TEF2*) and ubiquitin 10 (*UBQ10*). All eleven reference genes displayed a wide range of C_q _values in all samples, indicating that they expressed variably. The stability of these genes except for *RPL13 *was determined by three different descriptive statistics, geNorm, NormFinder and BestKeeper, which produced highly comparable results.

**Conclusion:**

Our study demonstrates that expression stability varied greatly between genes studied in peach. Based on the results from geNorm, NormFinder and BestKeeper analyses, for all the sample pools analyzed, *TEF2*, *UBQ10 *and *RP II *were found to be the most suitable reference genes with a very high statistical reliability, and *TEF2 *and *RP II *for the other sample series, while *18S rRNA*, *RPL13 *and *PLA2 *were unsuitable as internal controls. *GAPDH *and *ACT *also performed poorly and were less stable in our analysis. To achieve accurate comparison of levels of gene expression, two or more reference genes must be used for data normalization. The combinations of *TEF2*/*UBQ10/RP II *and *TEF2/RP II *were suggested for use in all samples and subsets, respectively.

## Background

Reverse transcriptase quantitative real-time polymerase chain reaction (RT-qPCR) has become a very powerful technique for detection and quantification of mRNA transcription levels of a selected gene of interest [1,2] due to its high sensitivity, specificity, reproducibility, no post-PCR processing and broad dynamic range [3], which allows a straightforward comparison between RNAs that differ widely in their abundance. To accurately quantify gene expression, many experimental variations should be taken into account, such as quality and amount of starting material, presence of inhibitors in different sample materials, primer design, and RNA extraction and retrotranscription efficiencies [1]. Therefore, selection of an appropriate normalization strategy is of crucial importance for the acquisition of biological meaningful data. Among several methods proposed so far [1,4], reference genes are the most frequently used to normalize RT-qPCR data and to control the experimental possible errors generated in the quantification of gene expressions, since the reference genes are exposed to the same preparation steps as the gene of interest.

An ideal reference gene, known as an internal control gene or as reference gene, should be expressed at a constant level across various conditions, such as developmental stages or tissue types, and its expression is assumed to be unaffected by experimental parameters [5,6]. Moreover, the reference gene and the target gene should have similar ranges of expression in the samples to be analyzed [7]. However, several recent studies have scrutinized the stability of commonly known reference genes like 18S ribosomal RNA (*18S rRNA*), β-actin (*ACT*), and glyceraldehydes-3-phosphate dehydrogenase (*GAPDH*) used for the quantification of mRNA expression, and have documented that these genes should be used with caution as internal controls, because they showed different behaviors under different experiment conditions [8-13]. The reason for these expressional variabilities may be that the reference genes not only participate in the basic cell metabolism but also take part in other cellular process [14,15]. If the chosen reference gene has a large expression fluctuation, the normalization will lead to erroneous gene expression profiles of the target gene of interest [16,17]. In addition, the choice of a suitable control gene will depend on the scope and nature of the experiment to be performed [6]. Therefore, the selection of the most stable gene or set of genes as internal controls is a critical step to control the variability between samples for quantitative gene expression studies with a sensitive RT-qPCR technique [8].

Recently, a growing number of published articles reflect the importance of reference genes and the need to validate them for each particular experimental model. Nevertheless, most of these studies mainly deal with human or animal tissues. However, only a few have concerned plants such as wheat [18], barley [19], rice [20-22], potato [13], soybean [23], grape [24], poplar [25], tomato [26,27], coffee [28] and *Arabidopsis thaliana *[29,30]. To the best of our knowledge, there have been no reports on the suitability of reference genes for RT-qPCR studies of differential expression of genes in peach (*Prunus persica *L. Batsch).

Peach fruit development and ripening are complex processes involving major changes in fruit metabolism [31]. Biochemical processes occur in a well-defined order under the control of a series of ripening-related genes leading to considerable changes in texture, pigmentation, taste and aroma [32]. The understanding of expression patterns of some key genes will help illuminate the mechanism involved in these processes in fleshy fruit and improve peach fruit quality and storage potential. Furthermore, studies of the molecular events associated with the ripening response of peach fruit to various exogenous regulators and melting and non-melting flesh genotypes may also help elucidate what contributes to fruit ripening.

The aim of this research was to select and evaluate the stability of 11 reference genes for the purpose of normalization in studying peach gene expression. Statistical methods implemented in geNorm [33], BestKeeper [34] and NormFinder [35] were used.

## Results

To identify the best reference genes for studies of peach gene expression, a RT-qPCR assay, based on SYBR green detection, was designed for the transcription profiling of the eleven genes (*18S rRNA*, *ACT*, *CYP2*, *TEF2*, *GAPDH*, *PLA2*, *RP II*, *RPL13*, *TUA*, *TUB *and *UBQ10*, Table 1). The specificity of the amplifications was confirmed by the presence of a single band of expected size for each primer pairs in agarose gels following electrophoresis (data not shown) and by the single-peak melting curves of the PCR products. The melting temperatures of all PCR products were given in Table 2. No primer dimers or other products were resulted from non-specific amplification. No signals were detected in the minus RT and no-template controls. Efficiency of PCR reactions varied from 1.671 for *RPL13 *to 1.828 for *ACT*, and correlation coefficients ranged between 0.9952 and 0.9996 for *RPL13 *and *RP II*, respectively (Table 2).

**Table 1:** Description of peach reference genes for RT-qPCR

Name^a^	Peach EST database accession number	*Arabidopsis *homolog locus^b^	*Arabidopsis *locus description	Function	Identities (%)
*18S rRNA*	TC1229	AT3G41768	18S ribosomal RNA	Cytosolic small ribosomal subunit, translation	97
*ACT*	TC1223	AT5G09810	Actin 2/7	Structural constituent of cytoskeleton	85
*CYP2*	TC1916	AT3G63400	Cyclophilin (CYP2)	Protein folding, RNA splicing	87
*TEF2*	TC3544	AT1G56070	Translation enlongation factor 2	Translation factor activity, nucleic acid binding	100
*GAPDH*	TC3113	AT1G13440	Glyceraldehyde-3-phosphate dehydrogenase	glycolysis	84
*PLA2*	DY636283	AT2G19690	Phospholipase A2 beta	Phospholipid metabolic process	71
*RP II*	TC1717	AT2G15430	RNA polymerase subunit	DNA-directed RNA polymerase activity, DNA binding	73
*RPL13*	TC5178	AT5G23900	60S ribosomal protein L13 (RPL13D)	Structural constituent of ribosome	100
*TUA*	TC2873	AT5G19780	Tublin alpha-5	Cytoskeleton structural protein	100
*TUB*	TC3624	AT1G75780	Tublin beta-1	Unidimensional cell growth, response to light stimulus	100
*UBQ10*	TC2782	AT4G05320	Ubiquitin 10	Protein modification process, protein binding	83

All peach ESTs were named based on similarity to *Arabidopsis *proteins determined via BLASTX. ^b ^Closest *Arabidopsis *homolog identified using TAIR BLAST [64]. AGI proteins database was queried with peach nucleotide sequences using BLASTX or in the case of 18S rRNA, *Arabidopsis *genome database with BLASTN.

**Table 2:** Primer sequences and amplicon characteristics for each of the 11 reference genes

Name	Forward Primer Sequence [5'-3']	Reverse Primer Sequence [5'-3']	Amplicon Size (bp)	Product TM (°C) ^#^	RT-qPCR Efficiency*	R^2^*
*18S rRNA*	TAGTTGGTGGAGCGATTTGTCTG	CTAAGCGGCATAGTCCCTCTAAG	114	88.2	1.796	0.9995
*ACT*	GTTATTCTTCATCGGCGTCTTCG	CTTCACCATTCCAGTTCCATTGTC	112	86.3	1.828	0.9993
*CYP2*	ACTCCAAAGCGTGTTAGAAAAGG	GTCTCTTCCACCATAACGATAGG	120	90.4	1.767	0.9986
*TEF2*	GGTGTGACGATGAAGAGTGATG	TGAAGGAGAGGGAAGGTGAAAG	129	88.3	1.818	0.9994
*GAPDH*	ATTTGGAATCGTTGAGGGTCTTATG	AATGATGTTGAAGGAAGCAGCAC	121	88.7	1.794	0.9994
*PLA2*	TCGCCGTCGTTATCTTCTCC	TACCGAATCCCAACAGAATTACAG	115	90.8	1.765	0.9995
*RP II*	TGAAGCATACACCTATGATGATGAAG	CTTTGACAGCACCAGTAGATTCC	128	85.3	1.800	0.9996
*RPL13*	GCAGCGACTGAAGACATACAAG	GGTGGCATTAGCAAGTTCCTC	103	87.7	1.671	0.9952
*TUA*	TTCTCTCTACTCATTCCCTCCTTG	GATTGGTGTATGTTGGTCTCTCG	117	83.9	1.812	0.9993
*TUB*	CCGAGAATTGTGACTGCCTTCAAG	AGCATCATCCTGTCTGGGTATTCC	124	88.2	1.826	0.9994
*UBQ10*	AAGGCTAAGATCCAAGACAAAGAG	CCACGAAGACGAAGCACTAAG	146	89.5	1.795	0.9994

^# ^The melting temperature of specific PCR product was calculated by Rotor-Gene V6.1 software (Corbett Research). * The RT-qPCR efficiency and correlation coefficients (R^2^) were determined with LinRegPCR software [67].

### Expression profiles of reference genes

**Figure 1:**
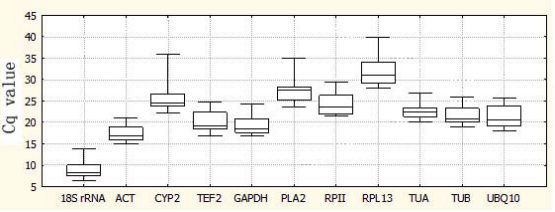
**RT-qPCR C_q _values for reference genes**. Expression data displayed as C_q _values for each reference gene in all peach samples. A line across the box is depicted as the median. The box indicates the 25th and 75th percentiles. Whiskers represent the maximum and minimum values.

Analysis of the raw expression levels across all samples identified some variation amongst reference genes (Figure 1). Quantification cycle (C_q_) [36] values for the 11 genes studied ranged from 8.2 to 30.9, while the majority of these values were between 18.6 and 24.6. The gene encoding *18S rRNA *was highly expressed compared to the protein coding genes, reaching threshold fluorescence after only 8.2 amplification cycles, whereas the C_q _average of all reference genes within the datasets was approximately 20.7 cycles. As a result, the *18S rRNA *transcript levels were around 5700-fold more abundant than the dataset's average. The least abundant transcripts were *PLA2 *and *RPL13*, with C_q _values of 27.6 and 30.9, respectively. The individual reference gene had different expression ranges across all studied RNA samples. *ACT*, *TUA *and *TUB *showed smaller gene expression variation (below 7 cycles) among studied reference genes, while *PLA2*, *CYP2 *and *RPL13 *had much higher expression variations (above 10 cycles). The wide expression ranges of the eleven tested reference genes confirmed that no single reference gene had a constant expression in different peach samples. Therefore, it is of utmost importance to select a reliable reference gene to normalize gene expression under a certain condition. Due to low expressed genes where C_q_s were obtained around cycles 30–35 can lead to large variability 34, the candidate *RPL13 *was discarded from subsequent tests.

### GeNorm analysis

**Figure 2:**
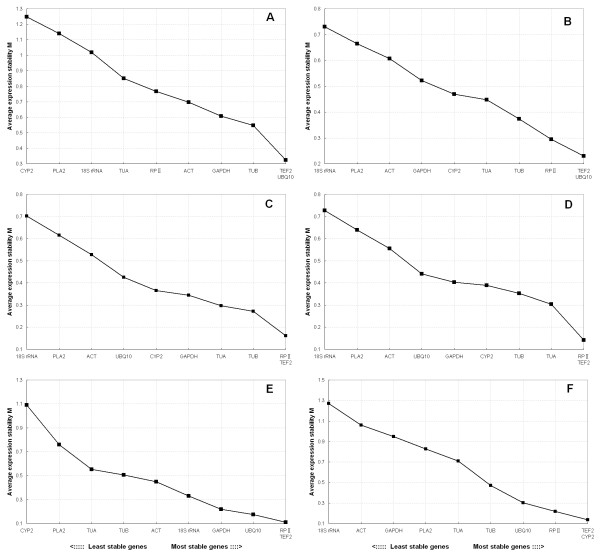
**Gene expression stability and ranking of the ten reference genes as calculated by geNorm**. Expression stability and ranking of ten reference genes calculated with geNorm in all the samples (A), fruit developmental series (B), different genotypes (C), different storage time series (D), different exogenous regulator treatments (E), different tissues (F). A lower average expression stability M value indicates more stable expression.

Gene expression stability (M) of these ten reference genes studied was calculated using the software geNorm [33,37]. The program is a Visual Basic application tool for Microsoft Excel and relies on the principle that the expression ratio of two perfect reference genes should be constant throughout the different experimental conditions or cell types. The M value is defined as the average pair-wise variation of a certain gene with all other tested reference genes, whereas the variation of this certain reference gene to another is determined as the standard deviation of the log2-transformed expression level ratios. The average expression M values of the 10 reference genes were plotted in Figure 2. The gene with the lowest M value is considered as the most stable expression, while the highest M value has the least stable expression. When all the samples were taken together, as shown in Figure 2A, the average expression stability value (M) of *TEF2 *and *UBQ10 *was the lowest, and that of *CYP2 *was the highest, indicating that *TEF2 *and *UBQ10 *had the most stable expression and that *CYP2 *was expressed most variably. *TEF2 *and *UBQ10 *were still the most stable genes, while *18S rRNA *was the one with the highest M value, suggesting that it was variably expressed in fruit developmental stages (Figure 2B). In the different genotype samples, *TEF2 *and *RP II *were the most stable genes, while *18S rRNA *was the least stable one (Figure 2C). The results remained very similar in the different storage time series, with the lowest M value for *RP II *and *TEF2 *and the highest M value for *18S rRNA *(Figure 2D). *TEF2 *and *RP II *were still expressed much more stably than the other reference genes in different exogenous regulator treatments, while *CYP2 *was the least stable reference gene as in all samples (Figure 2E). *CYP2 *and *TEF2 *were two best genes among the ten tested reference genes, while *18S rRNA *was the most variable one in different tissue samples of peach (Figure 2F).

**Figure 3:**
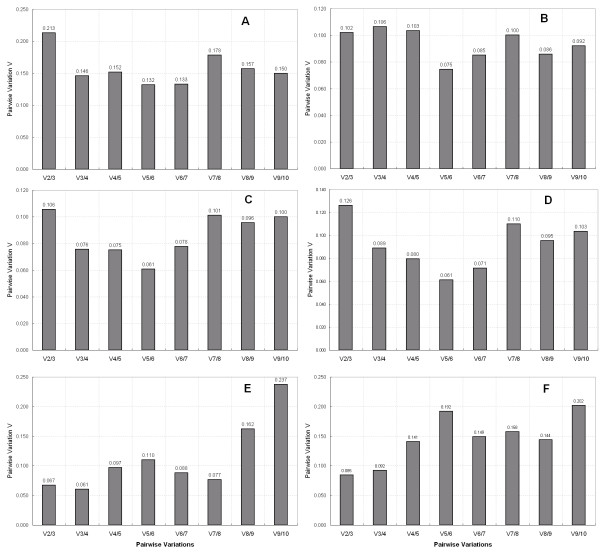
**Determination of the optimal number of reference genes**. Pairwise variation calculated by geNorm to determine the minimum number of reference genes for accurate normalization in all the samples (A), fruit developmental series (B), different genotypes (C), different storage time series (D), different exogenous regulator treatments (E), different tissues (F). Every bar represents change in normalization accuracy when stepwise adding more endogenous reference genes according to the ranking in Figure 2.

Although most authors agree in using only one single gene as an internal control for normalization, it has been suggested that the use of two or more reference genes for RT-qPCR studies might generate more reliable results [33,38]. To evaluate the optimal number of genes required for accurate normalization, pairwise variations Vn/Vn+1 between consecutively ranked normalization factors are calculated to determine the effect of adding the next reference gene in normalization. The normalization factors are defined by calculating the geometric mean of the 3 most stable gene relative quantities and stepwise inclusion of the other genes in the order of their expression stability. A large pairwise variation implies that the added gene has a significant effect on normalization and should be included for calculation of a reliable normalization factor [33]. As shown in Figure 3(B, C, D, E, F), the inclusion of the third reference gene did not contribute significantly to the variation of the normalization factor (V_3/4 _< 0.15). Based on the cut-off value of 0.15 proposed by geNorm program, below which the inclusion of an additional reference gene is not required, so the two most stable reference genes of each series subset would be sufficient for accurate normalization. When all the samples were taken together, the pairwise variation V_2/3 _was 0.213, higher than 0.15, while V_3/4 _was 0.146 (Figure 3A), indicating that the addition of the third reference gene was necessary to normalize gene expression. The 3 reference genes were *TEF2*, *UBQ10 *and *TUB *for this group samples. The recommended combinations of control genes of each sample series had mean stability values, M ≤ 1.0 and M ≤ 0.5, which are acceptable for heterogeneous and homogeneous sample panels, respectively [39]. Important to note is that the pairwise variation V and mean M values, calculated by the method of Exposito-Rodriguez *et al*. [27], for the combinations of *TEF2/UBQ10/RP II *and *TEF2/RP II *in all the sample pools and the series of fruit developmental and different tissue samples respectively, were all inside the ranges described as above (data not shown).

### NormFinder analysis

NormFinder, another VBA applet, uses a model-based approach for identifying the optimal normalization gene(s) among a set of candidates. More stable gene expression is indicated by lower average expression stability values. In this mathematical model, estimation of both intra- and inter-group variation and a separate analysis of the sample subgroups in expression levels are included into the calculation of a gene expression stability value [35]. In this sense, five sample-subgroups were established as geNorm analysis. Moreover, expression data were also combined into "vegetative" (stems, roots, leaves, flowers and fruit at different developmental stages) and "mature" (including fruit obtained from different exogenous regulator treatments and two cultivars at different storage time) sample-subgroups. At the same time, all samples with no subgroups and the other five series were analyzed using this approach as well. The results of the NormFinder analysis were shown in Table 3 and Additional file 1. It is noteworthy that definition of sample-subgroups had a notable effect on NormFinder output. However, the NormFinder output with different sample-subgroups and no subgroups exhibited two common features: 1) *TEF2*, *RP II *and *UBQ10 *showed a remarkable stability of their expression levels and were always classified among the top four positions; 2) *GAPDH*, *PLA2*, *ACT *and *18S rRNA *exhibited unstable expression profiles and were always included among the least stable reference genes. When it came to the other sample series, *TEF2 *was calculated to be the most stable single gene with a stability value of 0.007, while *18S rRNA *was the most variable one in different exogenous regulator treatments. The results were broadly similar to the series of different tissues, with the highest stability value for *TEF2 *and lowest stability value for *18S rRNA*. *RP II *was the most reliable gene in fruit developmental and different storage time series, while *18S rRNA *remained the most variable one. In different genotype samples, *TUB *was identified as the most stable gene and *PLA2 *was the least one, with stability values of 0.002 and 0.053, respectively.

**Table 3:** Ranking of candidate reference genes in order of their expression stability calculated by NormFinder

Ranking order	All samples			Fruit developmental series	Different genotypes	Different storage time series	Different regulator treatments	Different tissues
						
	No subgroups	2 subgroups	5 subgroups					
1	*TEF2*	*RP II*	*TEF2*	*RP II*	*TUB*	*RP II*	*TEF2*	*TEF2*
2	*UBQ10*	*TEF2*	*RP II*	*TUB*	*RP II*	*TUA*	*GAPDH*	*CYP2*
3	*RP II*	*CYP2*	*TUB*	*TEF2*	*TEF2*	*TUB*	*UBQ10*	*UBQ10*
4	*TUA*	*UBQ10*	*UBQ10*	*ACT*	*GAPDH*	*TEF2*	*RP II*	*RP II*
5	*TUB*	*TUA*	*CYP2*	*TUA*	*ACT*	*GAPDH*	*ACT*	*TUA*
6	*CYP2*	*TUB*	*TUA*	*UBQ10*	*TUA*	*ACT*	*PLA2*	*TUB*
7	*GAPDH*	*ACT*	*GAPDH*	*CYP2*	*CYP2*	*CYP2*	*TUB*	*PLA2*
8	*ACT*	*PLA2*	*ACT*	*GAPDH*	*UBQ10*	*UBQ10*	*CYP2*	*ACT*
9	*PLA2*	*GAPDH*	*PLA2*	*PLA2*	*18S rRNA*	*PLA2*	*TUA*	*GAPDH*
10	*18S rRNA*	*18S rRNA*	*18S rRNA*	*18S rRNA*	*PLA2*	*18S rRNA*	*18S rRNA*	*18S rRNA*

Details on stability values are available as additional file 1.

### BestKeeper analysis

BestKeeper, an Excel-based tool, estimates inter-gene relations of possible reference gene pairs by performing numerous pairwise correlation analyses using raw C_q _values of each gene. More important, all genes may be included in the calculation of the BestKeeper index, which can be used to rank the best reference genes because of stable reference genes showing a strong correlation with the BestKeeper index [34]. The results of the method analysis of the same data set were presented in Table 4 and Additional file 2. The 10 reference genes studied in our analysis correlated well one with another, if also compared with the BestKeeper index, except for *18S rRNA *in different tissue samples. Particularly strong inter-gene correlations were found for the four most stable reference genes in all the sample pools (r > 0.89), especially in the other five series (r > 0.95). The high Pearson's coefficients of correlation indicated that these gene pairs had very similar overall expression patterns. When the complete data set was analyzed, *TEF2*, *UBQ10*, and *RP II *had strong correlation with the BestKeeper index (r > 0.95), and ranked among the top four genes, in accordance with the corresponding NormFinder output, thus identifying these three genes as the most reliable reference genes for normalization. The result was identical to the series of different tissues, different genotypes, different regulator treatments and fruit developmental stages, because of *TEF2*, *UBQ10*, and *RP II *were still included among the 4 top-ranked reference genes. In different storage time series, only *TEF2 *and *RP II *were classified among the top four genes. *GAPDH*, *PLA2*, and *18S rRNA *consistently ranked poorly in the six series, and were identified as the least reliable reference genes.

**Table 4:** Ranking of the ten genes according to correlations between reference genes and BestKeeper index

Ranking order	All samples	Fruit developmental series	Different genotypes	Different storage time series	Different regulator treatments	Different tissues
1	*TEF2*	*TEF2*	*TEF2*	*TUA*	*RP II*	*CYP2*
2	*UBQ10*	*UBQ10*	*TUB*	*TEF2*	*TUB*	*RP II*
3	*RP II*	*TUB*	*RP II*	*RP II*	*TEF2*	*TEF2*
4	*TUA*	*RP II*	*UBQ10*	*ACT*	*UBQ10*	*UBQ10*
5	*CYP2*	*CYP2*	*TUA*	*CYP2*	*ACT*	*TUA*
6	*GAPDH*	*TUA*	*CYP2*	*UBQ10*	*CYP2*	*TUB*
7	*TUB*	*ACT*	*ACT*	*TUB*	*GAPDH*	*PLA2*
8	*ACT*	*18S rRNA*	*GAPDH*	*GAPDH*	*18S rRNA*	*GAPDH*
9	*PLA2*	*GAPDH*	*18S rRNA*	*18S rRNA*	*PLA2*	*ACT*
10	*18S rRNA*	*PLA2*	*PLA2*	*PLA2*	*TUA*	*18S rRNA*

Details on Pearson's correlation coefficients (r) are available as additional file 2.

## Discussion

The reliability of RT-qPCR data will be greatly improved by inclusion of a reference gene whose transcription level should be invariable in the different experimental conditions [4]. The present study is the first detailed survey on the stability of a large number of genes used as internal controls for RT-qPCR studies of differential expression of genes in peach.

Several approaches have been proposed to identify stability of gene expression and select the best reference genes in the context of the relevant experimental conditions [33-35,40-45], but to date, there is no consensus on which method should be used to examine reference gene expression stability. A comparison of different algorithms of reference gene selection allows a better evaluation of the most reliable controls and reduces the risk of artificial selection of co-regulated transcripts [46]. In order to select suitable reference gene(s) for accurate normalization, we compared three different statistical approaches, geNorm, NormFinder and BestKeeper to evaluate ten reference genes in peach.

The geNorm software is highly dependent on the assumption that none of the genes being analyzed are co-regulated as this would lead to an erroneous choice of optimum normaliser pair [35]. An obvious prediction about behavior of two co-regulated genes in the software is that they will occupy closed positions in the ranking [27]. In order to investigate whether the potential co-regulated genes *TUA *and *TUB *affected the outcome of our results, we removed one of them out of analysis and could not see any difference in the results, showing that in our data co-regulation did not affect the ranking of reference genes by stability. It should be worth mentioning that reference genes belonging to the same functional class that are not top-ranked by geNorm software in many previous studies [27]. Since it is very difficult to foresee common expression patterns, the stability of each reference gene expression was further assessed by NormFinder and BestKeeper that are less sensitive towards co-regulation of the reference genes.

The most prominent observation after completing the three analysis softwares was that each produced a different set of top ranked reference genes, and a fact that was not unexpected because the three programs based on different algorithms and analytical procedures. Generally, the analyses found that *TEF2*, *UBQ10 *and *RP II *were the most reliable internal controls for accurate normalization when looking at the expression data set as a whole, because these three genes were always classified among the 4 best performing reference genes except for *RP II *analyzed by geNorm in all the sample pools. For the other five series, *TEF2 *and *RP II *always ranked on top positions, exhibited stable expression patterns, and could serve as internal controls. On the other hand, *18S rRNA *and *PLA2 *ranked poorly based on all the three software programs, indicating that these two genes were not consistently expressed and should be avoided as internal controls when doing gene expression studies in our experimental setup.

*TEF2 *and *RP II *were abundantly and constantly transcribed in all of the peach samples. Indeed, these two genes are known to be required for elongation and mRNA transcription in eukaryotes, respectively [43,47]. So *TEF2 *and *RP II *remained continuously expressed over the different measured tissues and showed minimal changes in RNA transcription under different conditions. Regarding *UBQ10*, it was suggested to be an inappropriate internal control for RT-qPCR studies in different tissues at different developmental stages in rice [21] and soybean [23]. However, in an earlier study in *Arabidopsis *[29] and tomato [48], *UBQ10 *showed highly stable expression. But in the current study, results from all the three software analysis showed that *UBQ10 *underwent variation according to the experimental conditions. Consequently, it should be used with caution as an internal control. An ubiquitin tag is not only used to mark particular proteins for proteolytic elimination, but also has non-proteolytic functions [49] which may affect its level of expression in different plants. Based on the results from three software analysis, *CYP2*, stable in different peach tissue samples, was not the most stable in the other five series. Similarly, *CYP2 *was not among the best reference genes in any of the earlier analyses [13,24]. The reason may be that *CYP *expression is significantly regulated by development or exposure to certain stress inducers, such as ethephon, salicylic acid in plants [50]. Other reference genes, like *TUB*, *TUA*, and *PLA2 *displayed unacceptably variable expression patterns, limiting their use as internal controls. Surprisingly, *TUA *showed highly stable expression in tested tissue samples of poplar among the 10 reference genes [25]. Taken together, these results suggested that a reference gene with stable expression under a certain condition may not be suitable to normalize gene expression under another condition, that is to say, reliable reference genes are highly specific for a particular experimental situation, thus requiring a careful evaluation for every individual experimental setup.

The most striking result was the poor performance of the most popular reference genes. *GAPDH *has been the one that is widely used in many areas of research [9] and is one of the best reference genes for measuring the gene expression in many tissues [24,28,51]. However, there have been also previous examples of this gene leading to wrong results due to its lack of stability in specific experimental conditions [9,21]. In present analysis, *GAPDH *was not among the best reference genes between experimental groups. Reasons for those discrepancies may be that *GAPDH *not only acts as a component of the glycolytic pathway but also takes part in other processes as well. Thus, the expression profile of *GAPDH *might fluctuate according to the corresponding experimental conditions. Another most commonly used reference gene, *18S rRNA*, performed worst and were not among the more stable genes in our tests. The poor stability of *18S rRNA *in broomrape tissues was also found by Gonzalez-Verdejo *et al*. [52]. Previously, the *18S rRNA *gene was considered to be an ideal internal control in RT-qPCR analysis [53]. However, there are several arguments against the use of *18S rRNA *as an internal control. Its high abundance compared with target mRNA transcripts makes it difficult to subtract the baseline value in RT-qPCR data analysis accurately [33], and also makes it necessary to dilute the cDNA samples prior to real-time analysis, thus risking dilution errors [54]. Again, *18S rRNA *can not be used as a reference gene when reverse transcription reaction is carried out using oligo-dT primers or only mRNA is used as template [21]. Furthermore, *18S rRNA *synthesis is also regulated [55]. It is precisely for these reasons that *18S rRNA *has failed to replace the use of other reference genes [56]. *ACT*, the third mainly used reference gene, has been widely used as reference gene in gene expression studies in many organisms. Nevertheless, recent studies revealed that *ACT *did not satisfy certain basic requirements for application as an internal control [13,57]. Our analysis also showed that *ACT *was not the best reliable gene for comparative expression analysis. This may partly be explained by the fact that *ACT*, one of the major components of cytoplasmic microfilaments in eukaryotic cells, not only supports the cell and determines its shape but also participates in other cellular functions [56]. These results confirmed, once more, the need to evaluate reference genes in each experimental setting.

Earlier studies on the physiology of peach ripening have indicated that ethylene, abscisic acid (ABA), jasmonic acid (JA), 1-methylcyclopropene (1-MCP) and indole acetic acid (IAA) could modulate ripening [58-61]. However, the effects of those regulators on the expression of ripening-related genes, such as pectate lyase (*PL*), expansin (*EXP*) galactosidase (*GAL*), lipoxygenase (*LOX*), and so on, have not been elucidated in peach in detail. Moreover, the transcript levels of these genes in melting and non-melting flesh cultivars are quite variable. Studies of the molecular events associated with the ripening responses of fruit to various exogenous regulators and different genotypes will be beneficial in improving peach fruit quality and storage potential. In the present study, based on geNorm, NormFinder and BestKeeper methods, the most stable reference genes in the different cultivar and treatment samples were *TEF2 *and *RP II*. Analyses by geNorm applet suggested that the combination of the two genes was the optimal set of internal controls for studying differential gene expression in peach by RT-qPCR under the two conditions. Using the most reliable reference genes for normalization would be helpful to understand the molecular mechanisms involved in peach fruit ripening for different genotypes and regulator treatments.

## Conclusion

Our data showed that expression stability varied considerably between genes in different tissue samples and under different experimental conditions in peach. Using the software applications BestKeeper, geNorm and NormFinder, *TEF2*, *UBQ10 *and *RP II *appeared to be the three most suitable reference genes for all the sample pools, and *TEF2 *and *RP II *for the other series, while *18S rRNA*, *RPL13 *and *PLA2 *seemed to be unsuitable as internal controls. *GAPDH *and *ACT *also performed poorly and were less stable in our analysis. In order to get the most reliable results in peach gene profiling studies, more than one reference gene was recommended as internal controls for relative gene quantification. These results may provide a guideline for future works on gene expression in peach using RT-qPCR.

## Methods

### Plant materials and treatments

Tissues of Yuhua 1, a melting flesh peach genotype, were sampled from 8-year-old trees growing in national germplasm orchard of Institute of Horticulture of Jiangsu Academy of Agricultural Sciences, Nanjing, Jiangsu, China. Vegetative tissue samples, such as root, leaf and stem, were taken from young tissue; flowers were harvested at full bloom; and fruit at different developmental stages were taken at 2-week intervals after anthesis over the growing season. At each sampling time, plant materials except for fruits were frozen in dry ice after immediately harvesting transported to the laboratory at Nanjing Agricultural University and then stored at -70°C until total RNA was isolated.

Fruit of Jingyu peach, a non-melting flesh peach genotype, was from Institute of Forestry and Pomology, Beijing Academy of Agricultural and Forestry Sciences, China. At a stage equivalent to commercial ripeness, at about 9.56% and 12.16% soluble solids concentration (SSC) for fruits of Jingyu and Yuhua 1, respectively, free from visual symptoms of any disease or blemishes were chosen and directly stored at 25 ± 1°C for 6 days. For 1-methylcyclopropene (1-MCP) and ethylene treatments, Yuhua 1 fruits were sealed in two closed airtight containers, and 1 μl/L 1-MCP and 100 μl/L ethylene were injected into the two containers through a rubber septum, respectively. Fruits were incubated with 1-MCP or ethylene for 24 h at 25 ± 1°C and then containers were open to allow ripening in air in the same temperature conditions. Fruits of Yuhua 1 were also treated with ABA and IAA by dipping in 100 μM solutions in 0.2% Tween 80 for 20 min, respectively. In the JA treatment, Yuhua 1 fruits were dipped for 10 min in 50 mM methyl-jasmonate (MeJA) solution which also contained 0.2% Tween 80. After being dipped, fruits were dried and then allowed ripening at 25 ± 1°C in the air. Sampling from fruits of the two cultivars in normal room temperature was carried out daily for 6 days. Samples of Yuhua 1 fruit from different storage were used to evaluate the stability of 11 reference genes, while Jingyu fruit samples were only used to study the effect of different genotypes on stability values of 11 reference genes. Samples for different regulator treatments were taken for day 3, including one sample taken before treatment. All experiments were replicated three times with ten fruit as an experiment unit for gene expression studies. For all fruit samples epicarp and endocarp were excluded and the mesocarp was frozen in liquid nitrogen and stored at -70°C until further use.

### Total RNA extraction

Total RNA was isolated according to the method described by Meisel *et al*. [62]. Genomic DNA was eliminated by treating each sample with RNase-free DNase I (TaKaRa, Japan) according to the instructions manual. The concentration of isolated total RNA was calculated from absorbance at 260 nm with BioPhotometer (Eppendorf, Hamburg, Germany), the purity was verified by optical density (OD) absorption ratio OD_260 nm_/OD_280 nm _between 1.80 and 2.05, and OD_260 nm_/OD_230 nm _ranging from 2.00 to 2.60 and the integrity was evaluated by electrophoresis on ethidium bromide-stained 1.0% agarose gels. Intact rRNA subunits of 18S and 28S were observed on the gel and absence of smears indicating minimal degradation of the RNA.

### First strand cDNA synthesis

One microgram RNA was reverse-transcribed using the SYBR PrimeScript RT-PCR kit II (TaKaRa, Japan) for first-strand cDNA synthesis with 2.5 μM oligonucleotide dT primer and 5 μM random hexamer priming method according to the manufacturer's recommendations. Before transcription, RNA and primers were mixed and incubated at 70°C for 5 min followed by cooling on ice immediately. The first strand cDNA synthesis was started after adding transcription mixture at 37°C lasting 15 min for reverse transcriptase reaction. Finally, the PrimeScript Reverse Transcriptase was inactivated by heating the reaction mixture for 5 sec at 85°C. Each RNA sample was controlled for genomic DNA contamination without reverse transcriptase addition into cDNA synthesis mixture. All cDNA samples were stored at -20°C and diluted 1:10 with RNase-free water before being used as template in RT-qPCR analysis.

### Selection of peach sequences and primer design

Eleven genes were selected for investigation to identify the most stably expressed reference gene(s) to be used in RT-qPCR studies. This group of genes comprised several classical reference genes which are the most commonly used as internal control for expression studies, such as *GAPDH*, *18S rRNA *and *ACT*, the others based on previous reports [25,43]. The peach EST database [63] was queried with *Arabidopsis *protein sequences using TBLASTN to select peach homologs of genes commonly used as internal controls for gene expression analysis. The chosen peach ESTs were then used to query the *Arabidopsis *protein database using BLASTX [64] to obtain the description of peach reference genes. The reference genes evaluated are listed on Table 1, as are the corresponding accession numbers, *Arabidopsis *homolog locus, *Arabidopsis *locus description and main functions.

Primer pairs for RT-qPCR amplification were designed based on selected sequences using Beacon Designer 7.0 software (Premier Biosoft International, Palo Alto, California, USA) with a melting temperature between 60–62°C, 20–26 bp and about 50% GC content. Amplicon lengths were optimized to 103–146 bp to ensure optimal polymerization efficiency and minimize the impact of RNA integrity on relative quantification of gene expression [65]. MFOLD software [66] was subsequently used to evaluate the target sequences amplified by the primer pairs to avoid the formation of secondary structures at the site of primer binding. The primers were further used to query peach EST database with BLASTN to confirm the identity of the genes. Before RT-qPCR, each primer pair was tested via standard RT-PCR to check for size specificity of the amplicon by 2.5% agarose gel electrophoresis and ethidium bromide staining. In addition, target amplicons were sequenced to confirm specificity of the PCR products. The primer sequences, amplicon sizes, and melting temperatures of all PCR products were indicated in Table 2.

### RT-qPCR with SYBR green

RT-qPCR was performed using a Rotor-Gene 3000 (Corbett Robotics, Australia) and the SYBR Green Real-time Master Mix (TOYOBO, Japan). The PCR reaction volume was 20 μL containing 1.5 μL of diluted cDNA and 0.2 μM of each primer. Thermocycling conditions were set as an initial polymerase activation step for 2 min at 95°C, followed by 45 cycles of 15 sec at 94°C for template denaturation, 15 sec at 60°C for annealing and 20 sec at 72°C for extension and fluorescence measuration. Afterwards, a dissociation protocol with a gradient from 57°C to 95°C was used for each primer pair to verify the specificity of the RT-qPCR reaction and the absence of primer dimer. In addition, each PCR reaction included a reverse transcription negative control to check for potential genomic DNA contamination. Reagent contamination was also detected by a reaction mix without template. All samples were amplified in triplicates and the mean was used for RT-qPCR analysis.

### Data analysis

Expression levels of the tested reference genes were determined by the number of amplification cycles (C_q_) needed to reach a specific threshold level of detection. All amplification plots were analyzed with a threshold fluorescence value of 0.1 to obtain C_q _values using the Rotor-Gene software version 6.1 (Corbett Research). The PCR efficiency showed in Table 2 was calculated for each gene with LinRegPCR program [67] from raw fluorescence data taken from the Rotor-Gene 3000 detection system. Results from the LinRegPCR and Rotor-Gene software were imported into Microsoft Excel and transformed to relative quantities using the comparative C_q _method and specific efficiencies for each gene [68]. The data obtained were converted into correct input files, according to the requirements of the software, and analyzed using three different VBA applets, geNorm (version 3.4) [33,37], NormFinder (version 0.953) [35,69] and BestKeeper (version 1.0) [34,70].

## Authors' contributions

ZT performed all the experimental procedures, data analysis, draft the manuscript and was the primary author of the manuscript. ZG and JZ assisted in manuscript revising and provided helpful discussions. FW performed the sample preparation and participated in tables and figures drawing. ZZ conceived and supervised the research, revised the manuscript and provided financial support. All authors read and approved the final manuscript.

## Supplementary Material

Additional file 1 **Stability values of reference genes calculated by NormFinder**. File showing the stability values of the ten selected candidate reference genes calculated by NormFinder. Click here for file

Additional file 2 **Inter-gene relations and correlations between the reference genes and the BestKeeper index**. The file shows pairwise correlation analyses were performed based on the C_q _values of the ten reference genes. Pearson's correlation coefficients (r) are shown. All the correlations are over the significance threshold (p = 0.05). Click here for file
