# Supplementary material for: Selection of reliable reference genes for gene expression studies in peach using real-time PCR
Source: BMC Mol Biol. 2009 Jul 20;10:71. doi: 10.1186/1471-2199-10-71 (PMC3224724; doi:10.1186/1471-2199-10-71)
Supplement: Additional file 1 — Stability values of reference genes calculated by NormFinder. File showing the stability values of the ten selected candidate reference genes calculated by NormFinder. [file 1471-2199-10-71-S1.pdf]

Gene name	All samples			Fruit developmental series	Different genotypes	Different storage time series	Different regulator treatments	Different tissues
	No subgroups	2 subgroups	5 subgroups					
18S rRNA	0.140	0.098	0.056	0.101	0.051	0.087	0.162	0.140
ACT	0.048	0.057	0.020	0.023	0.023	0.023	0.025	0.060
CYP2	0.045	0.028	0.015	0.028	0.030	0.028	0.058	0.015
GAPDH	0.047	0.064	0.018	0.036	0.021	0.023	0.007	0.068
PLA2	0.055	0.061	0.021	0.054	0.053	0.056	0.035	0.056
RPII	0.037	0.017	0.010	0.005	0.002	0.003	0.024	0.033
TEF2	0.019	0.027	0.007	0.020	0.005	0.020	0.006	0.010
TUA	0.042	0.044	0.015	0.024	0.026	0.009	0.060	0.034
TUB	0.043	0.047	0.012	0.008	0.002	0.014	0.052	0.050
UBQ10	0.027	0.039	0.013	0.026	0.033	0.031	0.007	0.031
