# Supplementary material for: Selection of reliable reference genes for gene expression studies in peach using real-time PCR
Source: BMC Mol Biol. 2009 Jul 20;10:71. doi: 10.1186/1471-2199-10-71 (PMC3224724; doi:10.1186/1471-2199-10-71)
Supplement: Additional file 2 — Inter-gene relations and correlations between the reference genes and the BestKeeper index. The file shows pairwise correlation analyses were performed based on the Cq values of the ten reference genes. Pearson's correlation coefficients (r) are shown. All the correlations are over the significance threshold (p = 0.05). [file 1471-2199-10-71-S2.pdf]

All samples

	18S rRNA	*ACT*	*CYP2*	*TEF2*	*GAPDH*	*PLA2*	*RPII*	*TUA*	*TUB*	*UBQ10*
ACT	0.645	-	-	-	-	-	-	-	-	-
CYP2	0.855	0.778	-	-	-	-	-	-	-	-
TEF2	0.788	0.934	0.870	-	-	-	-	-	-	-
GAPDH	0.609	0.925	0.841	0.945	-	-	-	-	-	-
PLA2	0.921	0.643	0.924	0.815	0.722	-	-	-	-	-
RPII	0.685	0.899	0.891	0.928	0.931	0.789	-	-	-	-
TUA	0.789	0.789	0.893	0.908	0.849	0.859	0.939	-	-	-
TUB	0.593	0.970	0.761	0.955	0.964	0.657	0.926	0.830	-	-
UBQ10	0.732	0.939	0.836	0.992	0.959	0.784	0.933	0.899	0.972	-
BestKeeper	0.851	0.906	0.939	0.981	0.926	0.893	0.950	0.943	0.912	0.967

Fruit developmental series

	18S rRNA	*ACT*	*CYP2*	*TEF2*	*GAPDH*	*PLA2*	*RPII*	*TUA*	*TUB*	*UBQ10*
ACT	0.982	-	-	-	-	-	-	-	-	-
CYP2	0.933	0.964	-	-	-	-	-	-	-	-
TEF2	0.960	0.990	0.988	-	-	-	-	-	-	-
GAPDH	0.844	0.903	0.980	0.951	-	-	-	-	-	-
PLA2	0.915	0.896	0.939	0.927	0.894	-	-	-	-	-
RPII	0.926	0.962	0.993	0.991	0.977	0.946	-	-	-	-
TUA	0.916	0.937	0.993	0.972	0.977	0.965	0.989	-	-	-
TUB	0.947	0.979	0.985	0.997	0.955	0.938	0.996	0.975	-	-
UBQ10	0.942	0.980	0.992	0.998	0.968	0.924	0.996	0.978	0.997	-
BestKeeper	0.959	0.981	0.993	0.997	0.959	0.949	0.995	0.986	0.995	0.996

Different genotypes

	18S rRNA	*ACT*	*CYP2*	*TEF2*	*GAPDH*	*PLA2*	*RPII*	*TUA*	*TUB*	*UBQ10*
ACT	0.988	-	-	-	-	-	-	-	-	-
CYP2	0.965	0.979	-	-	-	-	-	-	-	-
TEF2	0.965	0.975	1.000	-	-	-	-	-	-	-
GAPDH	0.957	0.981	0.997	0.995	-	-	-	-	-	-
PLA2	0.964	0.914	0.906	0.913	0.879	-	-	-	-	-
RPII	0.976	0.979	0.997	0.998	0.990	0.934	-	-	-	-
TUA	0.993	0.988	0.989	0.989	0.981	0.953	0.996	-	-	-
TUB	0.981	0.982	0.996	0.997	0.989	0.940	1.000	0.998	-	-
UBQ10	0.981	0.993	0.996	0.994	0.995	0.917	0.995	0.995	0.996	-
BestKeeper	0.987	0.990	0.991	0.999	0.989	0.939	0.997	0.994	0.998	0.995

Different storage time series

	18S rRNA	*ACT*	*CYP2*	*TEF2*	*GAPDH*	*PLA2*	*RPⅡ*	*TUA*	*TUB*	*UBQ10*
ACT	0.979	-	-	-	-	-	-	-	-	-
CYP2	0.982	1.000	-	-	-	-	-	-	-	-
TEF2	0.972	0.998	0.997	-	-	-	-	-	-	-
GAPDH	0.952	0.994	0.993	0.996	-	-	-	-	-	-
PLA2	0.972	0.934	0.937	0.937	0.901	-	-	-	-	-
RPⅡ	0.968	0.994	0.993	0.999	0.992	0.945	-	-	-	-
TUA	0.991	0.995	0.996	0.994	0.981	0.966	0.993	-	-	-
TUB	0.964	0.991	0.990	0.998	0.991	0.944	1.000	0.991	-	-
UBQ10	0.964	0.997	0.996	1.000	0.998	0.927	0.998	0.990	0.997	-
BestKeeper	0.984	0.995	0.993	0.998	0.989	0.954	0.997	0.999	0.990	0.992

Different regulator treatments

	18S rRNA	*ACT*	*CYP2*	*TEF2*	*GAPDH*	*PLA2*	*RPⅡ*	*TUA*	*TUB*	*UBQ10*
ACT	0.986	-	-	-	-	-	-	-	-	-
CYP2	0.973	0.998	-	-	-	-	-	-	-	-
TEF2	0.982	1.000	0.999	-	-	-	-	-	-	-
GAPDH	0.968	0.996	0.999	0.998	-	-	-	-	-	-
PLA2	0.991	0.961	0.942	0.955	0.932	-	-	-	-	-
RPⅡ	0.977	0.999	1.000	1.000	0.999	0.947	-	-	-	-
TUA	0.915	0.962	0.977	0.968	0.976	0.883	0.974	-	-	-
TUB	0.985	0.999	0.998	0.999	0.995	0.963	0.998	0.970	-	-
UBQ10	0.989	1.000	0.996	0.999	0.994	0.963	0.997	0.954	0.997	-
BestKeeper	0.991	0.996	0.995	0.998	0.992	0.970	0.999	0.959	0.998	0.997

Different tissues

	18S rRNA	*ACT*	*CYP2*	*TEF2*	*GAPDH*	*PLA2*	*RPⅡ*	*TUA*	*TUB*	*UBQ10*
ACT	0.404	-	-	-	-	-	-	-	-	-
CYP2	0.449	0.863	-	-	-	-	-	-	-	-
TEF2	0.424	0.880	0.999	-	-	-	-	-	-	-
GAPDH	0.089	0.967	0.928	0.937	-	-	-	-	-	-
PLA2	0.789	0.575	0.903	0.891	0.680	-	-	-	-	-
RPⅡ	0.433	0.857	0.998	0.996	0.936	0.893	-	-	-	-
TUA	0.715	0.575	0.905	0.889	0.728	0.963	0.914	-	-	-
TUB	0.156	0.974	0.951	0.961	0.992	0.729	0.951	0.744	-	-
UBQ10	0.405	0.887	0.999	1.000	0.945	0.881	0.997	0.885	0.966	-
BestKeeper	0.504	0.827	0.998	0.995	0.904	0.928	0.996	0.930	0.929	0.993
